# Association of leukocyte telomere length and HbA1c with post-COVID-19 syndrome in type 2 diabetes: a cross-sectional pilot study

**DOI:** 10.3389/fmed.2025.1628156

**Published:** 2025-08-29

**Authors:** Anton Matviichuk, Dmytro Krasnienkov, Viktoriia Yerokhovych, Yeva Ilkiv, Veronika Korcheva, Oleksandr Gurbych, Anna Shcherbakova, Pavlina Botsun, Tetyana Falalyeyeva, Oksana Sulaieva, Nazarii Kobyliak

**Affiliations:** ^1^Endocrinology Department, Bogomolets National Medical University, Kyiv, Ukraine; ^2^Laboratory of Epigenetics, Institute of Gerontology Academy of Medical Sciences of Ukraine, Kyiv, Ukraine; ^3^Blackthorn AI, Ltd., London, United Kingdom; ^4^Taras Shevchenko National University of Kyiv, Kyiv, Ukraine; ^5^Department of Artificial Intelligence Systems, Lviv Polytechnic National University, Lviv, Ukraine; ^6^Medical Laboratory CSD, Kyiv, Ukraine; ^7^Department of Pathology, Kyiv Medical University, Kyiv, Ukraine

**Keywords:** telomere length, type 2 diabetes, post-COVID-19, long COVID-19, HbA1c, biomarkers, aging, machine learning

## Abstract

**Introduction:**

Leukocyte telomere length is considered a promising prognostic marker associated with COVID-19 severity, adverse outcomes (hospital admission, need for critical care, and respiratory support), and mortality. However, the contribution of telomere length to post-COVID-19 syndrome (PCS) development is unclear.

**Aim:**

This study aimed to evaluate the association between telomere shortening and the course of PCS in patients with type 2 diabetes (T2D) and to determine whether telomere length is linked to clinical phenotype, gender, and biological age.

**Materials and methods:**

In this cross-sectional study, 66 T2D patients who had recovered from COVID-19 were enrolled. Patients were divided into two groups depending on PCS development: the PCS group (*n* = 44) and patients who did not develop PCS (*n* = 22) within 6 months after COVID-19 infection. Relative telomere length was determined using the standardized method proposed by Cawthon et al. A range of machine learning models was developed for PCS prediction. These models underwent training utilizing a cross-validation approach, as well as internal validation.

**Results:**

We observed a significantly lower mean of telomere length in T2D patients with PCS as compared to those without it (1.09 ± 0.19 and 1.28 ± 0.24; *p* = 0.001). In the sub-analysis, shorter telomeres were observed in female patients and patients of older age in both groups. The mean telomere length did not differ significantly among clinical phenotypes of PCS (*p* = 0.193). The best model generated for PCS prediction was the gradient boosting machine (GBM), which achieved an AUC of 0.753. The most influential variables across the top 10 models included telomere length, HbA1c, vitamin D_3_, waist circumference, ApoA1, C peptide, ApoB, COVID-19 severity, duration of T2D, IL-6, cholesterol, BMI, and age. Leukocyte telomere length and HbA1c exhibited significantly greater impact than other features.

**Conclusion:**

Shorter telomere length and higher HbA1c levels were significantly associated with the presence of PCS in our cohort of individuals with T2D. These factors may represent potential biomarkers that warrant further investigation.

## Introduction

The recent outbreak of severe acute respiratory syndrome caused by coronavirus 2 (SARS-CoV-2) and the related coronavirus infection (COVID-19), in which inflammation plays a critical role, has gripped the international community and raised widespread public health concern (1, 2). Notably, obesity and type 2 diabetes (T2D) were the most significant risk factors for severe forms of COVID-19, provoking various complications and a high death rate (3). Post-COVID-19 syndrome (PCS; long COVID-19, post-acute COVID-19, and long-term effects of COVID-19) became an emerging health problem in people recovering from COVID-19 infection within the past 3–6 months and is characterized by a set of symptoms, including fatigue, muscle pain, cough, drowsiness, headaches, and more (4). Currently, the bidirectional association between diabetes and long-COVID-19 has been discussed (5–7). Some evidence suggests that diabetes may be a risk factor for the development of PCS (6). Recent data also indicate that new-onset diabetes may be a complication of COVID-19 and represent the metabolic clinical phenotype of PCS (8, 9). However, the particular association between T2D and PCS are still under debate.

Studying the genetic code of aging is a very relevant topic in current times (10). The physiological processes of aging and the impact of pathological changes may be based on the length and quality of telomeres, which maintain chromosomal stability and prevent their degradation (11). The leukocyte telomere can be a surrogate reflection of a genetic clock (12). Telomeres shorten along with cell proliferation, so telomere length negatively correlates with cell replication numbers, reflecting a biological age (13).

Reduced telomere length and their attrition are associated with an increased incidence of cardiovascular, respiratory, digestive, and musculoskeletal diseases (14–16). Telomere shortening is observed in people with acute and chronic diseases, unhealthy habits, stress, and intoxication (17–20). It has also been proven that lifestyle modification, diet, and some medications can increase chromosomal length, thereby preventing the disease (21). It was also found that telomere length is shorter in people with diabetes than in individuals of the same age without metabolic disorders (22). This phenomenon was illustrated in both *β*-cells and peripheral blood leukocytes and inversely correlated with insulin resistance progression (23–25). Therefore, telomere length was assumed to be a candidate biomarker for predicting T2D progression.

Notably, telomere shortening is also a marker of COVID-19 severity and is linked to mortality (21). Naturally, physiological aging causes a decrease in telomere size, a decline in cell proliferation, and exhaustion of the reparative capabilities (26). It is believed that older people are more susceptible to coronavirus disease at the receptor level due to the damage or shortening of telomeres. This results in the expression of angiotensin-converting enzyme 2 (ACE2) and DNA damage response (DDR), which leads to increased disease severity, more frequent complications, hospitalizations, and mortality (26). Another argument proving the role of telomeres in the aging process was demonstrated in a study on mice with severe viral pneumonia. Macrophages with shortened telomeres were found in lung tissue, and their accelerated senescence was caused by mitochondrial distress and abnormal activation of the STING and NLRP3 inflammatory pathways. Such results may explain the concept of innate immune aging and encourage the study of the role of senolytics for symptomatic support of severe viral pneumonia in the elderly (27).

Recent data regarding telomere length in patients with PCS are very scarce and limited to several reports, which demonstrate its shortening in cases with pulmonary sequelae (28–30). Although COVID-19 and T2D affect telomeres, there is a gap in data concerning a link between PCS, T2D, and telomere length.

The current study aimed to investigate the association between leukocyte telomere length and clinical features, personal history, inflammatory markers, and metabolic profile in patients with T2D suffering from PCS with different clinical phenotypes.

## Materials and methods

### Study design

In this cross-sectional pilot study, 66 patients with T2D were recruited from the University Hospitals of Bogomolets National Medical University and Kyiv City Clinical Endocrinology Center using a convenience sampling method. As this was an exploratory pilot study, a formal *a priori* sample size calculation was not performed; the sample size was based on the availability of eligible patients who consented to participate during the recruitment period.

The inclusion criteria were age over 18 years and the presence of T2D and COVID-19 infection confirmed by a positive Reverse transcription polymerase chain reaction (RT-PCR) test (Seegene, South Korea). Next, patients were divided into two groups depending on PCS presence: PCS group (main group, *n* = 44) and patients who did not develop PCS (comparison group, *n* = 22) for up to 6 months after COVID-19 infection. PCS was defined according to the WHO criteria as the continuation or development of new symptoms more than 3 months after the initial SARS-CoV-2 infection. Patients with the following PCS clinical phenotypes were included in the study: a predominance of fatigue and/or pain, mental disorders, cardiorespiratory symptoms, and metabolic-associated symptoms (which are defined as fluctuating, increasing, persistent, or relapsing pre-existing chronic complications or the natural course of T2D). The exclusion criteria included type 1 diabetes, new-onset diabetes due to COVID-19 or secondary diabetes, and active malignancy.

The following clinical and demographic data were collected: age, gender, anthropometric indicators, T2D duration and age of onset, T2D complications, history of COVID-19, COVID-19 severity and treatment, PCS phenotype and symptoms, duration of PCS, and hypoglycemic therapy. According to the WHO classification, COVID-19 was categorized into mild, moderate, or severe infection. Mild COVID-19 was defined as respiratory symptoms without evidence of pneumonia or hypoxia, while moderate or severe infection required the presence of clinical and radiological evidence of pneumonia. In moderate cases, SpO_2_ was ≥90% on room air, while one of the following was required to define the severe cases: respiratory rate >30 breaths/min or SpO_2_ < 90% on room air (31, 32).

Anthropometric data, including weight and height, were measured to the nearest 100 g and 0.5 cm, respectively. Body mass index (BMI) was calculated as body weight in kilograms divided by the square of the participant’s height in meters (weight/height^2^). Waist (narrowest diameter between xiphoid process and iliac crest) circumference (WC) was also measured.

### Laboratory measurements

In the main group, the blood samples were drawn from patients during hospital admission in the active PCS phase. In the comparison group, blood was collected during the standard follow-up visit in the same period. The venous blood was collected after a 12-h overnight fasting in EDTA tubes (final concentration of 0.1% EDTA) and serum separator gel blood collection tubes.

The blood samples were analyzed for complete blood count (Sysmex 550, Japan), basic metabolic panel (AU-480, Beckman Coulter, United States), HbA1c level (G8), high sensitivity C-reactive protein (hs-CRP), apolipoprotein A and apolipoprotein B (AU-480, Beckman Coulter, United States), vitamin D, and IL-6 (*Maglumi X8*, China) in an ISO 15189 certified laboratory according to the manufacturer’s instructions and standard operating procedures (SOPs).

### Telomere length determination

DNA samples were isolated from peripheral blood mononuclear cells using the phenol–chloroform purification method. The quality of DNA preparations was determined by spectral characteristics, using an ND-1000 spectrophotometer (NanoDrop, United States) in the range of λ220–λ300.

To determine the relative telomere length, a standardized method of quantitative monochrome multiplex polymerase chain reaction in real time (MM–qPCR) was used, which was proposed by Cawthon (33). This reaction was performed in the presence of SYBR Green I intercalating dye using HOT FIREPol® Probe qPCR Mix (Solis BioDyne, Estonia), in accordance with the manufacturer’s recommendations, on a Bio-Rad Chromo4 amplifier. For each sample, 10 ng DNA was used along with a pair of primers telg 5′–ACACTAAGGTTTGGGTTTGGGTTTGGGTTTGGGTTAGTGT–3′ and telc 5′–TGTTAGGTATCCCTATCCCTATCCCTATCCCTATCCCTAACA–3′ (final concentration, 450 nmol each), as well as a pair of albumin primers albu 5′–CGGCGGCGGGCGGCCGGGGCTGGGCGGCTTCATCCACGTTCACCTTG–3′ and albd 5′–GCCCGGCCCGCCGCGCCCGTCCCGCCGGAGGAGAAGTCTGCCGTT–3′ (final concentration of each, 250 nmol). These were added to 18 μL of the reaction mixture containing betaine at a final concentration of 1 M. All experimental DNA samples were analyzed in triplicate. Cy0 alternatives to threshold cycles, as in Guescini et al., were used instead of threshold cycle (Ct) (34). Normalized data were calculated using standard curves generated from serial dilutions (1, 1/3, 1/9, 1/27) of a randomly selected sample. Separate standard curves were created for the telomeric signal and the single-copy gene signal. Cy0 values were used with a standard curve to determine telomeric DNA amount (T) relative to reference DNA; data for albumin DNA(S) amount were obtained in a similar manner. Relative telomere length was determined as T/S ratios.

### Statistical analysis

Statistical analysis was performed using standard software SPSS version 20.0 (SPSS, Inc., Chicago, Illinois, United States) and GraphPad Prism, version 6.0 (GraphPad Software, Inc., La Jolla, CA, United States). Data distribution was analyzed using the Kolmogorov–Smirnov normality test. All continuous values were expressed as mean±SD, and categorical variables were presented as percentages. For comparison of telomere length between different clinical phenotypes of PCS, analysis of variance (ANOVA) was performed. The independent samples *t*-test was used to compare differences between groups. For comparisons of categorical variables, we conducted a χ^2^ test. The relationship between relative telomere length and main parameters was assessed using univariate Pearson’s correlation analysis. Finally, univariate logistic regression analysis was applied to assess the association between potential risk factors, including telomere length, and the presence of PCS in patients with T2D.

### Model development

We used the open-source H2O.ai autoML package for Python (35), enabling utilization on a local device to ensure patient data confidentiality. This package facilitates the training and cross-validation of various common machine learning algorithms, including gradient boosting machine (GBM), extreme gradient boosting (XGBoost), general linear models (GLMs), extremely randomized trees (XRT), distributed random forest (DRF), and deep learning (DL). Moreover, the package generates two types of stacked ensemble models–one utilizing all previously trained models and another incorporating the best model from each model family. Further details regarding the construction of each model and the hyperparameters tuned via autoML are available in the documentation provided by H2O.ai (35). Prior to utilization in model training, all continuous features underwent quantile transformation. Afterward, we used autoML to train 50 models using the 5-fold cross-validation, ranking them based on performance. Permutation importance and Shapley additive explanations (SHAP) were computed for the top model. To determine the most influential variables, we used an aggregated feature importance approach. First, permutation feature importance was calculated for each variable within our top 10 performing classification models. The variables were then ranked according to their median importance score across all 10 models.

GBMs are ensemble learning methods that combine the predictions of multiple weak learners to improve accuracy and robustness. In our case, GBMs performed best, likely because they effectively handle small datasets with many features, leveraging their ability to capture complex interactions and patterns without overfitting.

To assess the diagnostic accuracy of the developed models for predicting PCS, we used receiver operating characteristic (ROC) curves with the analysis of the area under the ROC curve (AUROC). AUROC values close to 1.0 indicated high diagnostic accuracy. Optimal cutoff values were chosen to maximize the sum of sensitivity and specificity (36).

## Results

### Characteristics of the study participants

Overall, we examined 66 patients with T2D who had COVID-19 in 2023. The main group (*n* = 44) experienced clinical signs of PCS of varying duration and severity. The comparison group (*n* = 22) did not report the development of PCS for more than 6 months after COVID-19 infection. The mean age was comparable between groups (62.09 ± 9.46 vs. 62.45 ± 11.45 years, *p* = 0.896). Anthropometric, clinical, and laboratory parameters are presented in Table 1. It should be noted that patients with PCS had higher weight, WC, and BMI; however, the differences between groups were not statistically significant. The mean duration of T2D in patients with PCS was 10.13 ± 6.93 years, which was comparable to the comparison group (10.68 ± 8.06, *p* = 0.721; Table 1).

**Table 1:** General participant and COVID-19 characteristics.

Parameter	PCS absent (*n* = 22)	PCS present (*n* = 44)	*p*
Age, years	62.09 ± 9.46	62.45 ± 11.14	0.896
T2D duration, years	10.81 ± 7.90	10.13 ± 6.93	0.721
Weight, kg	86.59 ± 12.12	87.45 ± 15.45	0.820
BMI, kg/m^2^	29.63 ± 4.60	30.43 ± 5.69	0.567
Waist circumference, cm	98.22 ± 12.61	99.18 ± 12.40	0.770
Metformin, % (n)	72.7 (16)	75.0 (33)	0.842
SUs, % (n)	27.3 (6)	34.1 (15)	0.575
DPP-4 inhibitors, % (n)	4.5 (1)	2.3 (1)	0.612
GLP-1 agonists, % (n)	4.5 (1)	2.3 (1)	0.612
SGLT-2 antagonists, % (n)	4.5 (1)	15.9 (7)	0.182
Insulin human, % (n)	9.1 (2)	11.4 (5)	0.777
Insulin analogs, % (n)	27.3 (6)	22.7 (10)	0.685
Diet only, % (n)	-	2.3 (1)	0.476
Monotherapy, % (n)	27.3 (6)	36.4 (16)	0.720
Combination of ADD, % (n)	31.8 (7)	38.6 (17)	0.587
Combination of ADD with insulin, % (n)	27.3 (6)	22.7 (10)	0.685
Diabetic nephropathy, % (n)	9.1 (2)	27.3 (12)	0.089
Diabetic peripheral neuropathy, % (n)	68.2 (15)	70.5 (31)	0.850
Diabetic autonomic neuropathy, % (n)	-	4.5 (2)	0.310
Diabetic retinopathy, % (n)	22.7 (5)	31.8 (14)	0.442
Diabetic foot, % (n)	13.6 (3)	9.1 (4)	0.572
No complication, % (n)	27.3 (6)	25.0 (11)	0.842
Myocardial infarction, % (n)	-	4.5 (2)	0.310
Stroke, % (n)	9.1 (2)	6.8 (3)	0.742
HbA1c, %	7.83 ± 1.26	8.81 ± 1.55	**0.012**
Total cholesterol, mmol/L	5.81 ± 1.69	5.57 ± 1.66	0.584
Apolipoprotein B, g/L	0.95 ± 0.31	0.94 ± 0.36	0.946
Apolipoprotein A1, g/L	1.79 ± 0.54	1.60 ± 0.41	0.119
Vitamin D_3_, ng/mL	21.89 ± 12.67	17.43 ± 8.26	0.091
hs-CRP, mg/L	4.14 ± 2.34	5.14 ± 4.32	0.315
IL-6, pg/mL	2.82 ± 2.44	3.72 ± 3.43	0.278
COVID-19 characteristics			
No treatment, % (n)	22.7 (5)	13.6 (6)	0.350
Supplements/NSAIDs, % (n)	77.3 (17)	86.4 (38)	0.350
Antibiotics, % (n)	40.9 (9)	54.5 (24)	0.296
O_2_ therapy, % (n)	9.1 (2)	27.3 (12)	0.089
Steroids, % (n)	13.6 (3)	36.4 (16)	0.055
Mechanical ventilation, % (n)	4.5 (1)	4.5 (2)	0.999
COVID-19 severity (WHO), % (n)			
Mild, % (n)	72.8 (16)	43.2 (19)	0.161
Moderate without hospitalization, % (n)	13.6 (3)	29.5 (13)	
Moderate with hospitalization, % (n)	9.1 (2)	18.2 (8)	
Severe, % (n)	4.5 (1)	9.1 (4)	

The data are presented as percentages (frequency) and mean±SD; BMI, body mass index; SUs, sulfonylureas; ADD, anti-diabetic drugs; PCS, post-COVID-19 syndrome. NSAIDs, non-steroidal anti-inflammatory drugs; WHO, World Health Organization. The bold indicates significant changes.

Among the most common anti-diabetic drugs (ADD) prescribed to patients were biguanides and sulfonylureas (SUs). More than 70% of patients in both groups received metformin (*p* = 0.842). No significant differences in prescribed ADD were found between groups. However, the 2-fold higher percentages of patients without PCS were treated with DPP-4 inhibitors and GLP-1 agonists (4.5% vs. 2.3%; *p* = 0.612). Furthermore, patients without PCS more often received insulin analogs (27.3% vs. 22.7%; *p* = 0.685), whereas patients with PCS more often received SUs (34.1% vs. 27.3%; *p* = 0.575). However, these treatment differences were not statistically significant between groups.

The chronic T2D complication profile is presented in Table 1. A higher prevalence of all chronic complications, except diabetic foot, was observed in patients with PCS. In addition, diabetic nephropathy was three times more common in the PCS group than in patients without PCS (27.3% vs. 9.1%; *p* = 0.089). This may explain the more frequent prescriptions of SGLT-2 antagonists in the observed groups (4.5% vs. 15.9%; *p* = 0.182).

Analysis of main laboratory parameters revealed that patients with PCS demonstrated worse glycemic control and higher levels of pro-inflammatory biomarkers. For instance, the level of HbA1c was significantly higher in PCS patients (8.81 ± 1.55%) than in the comparison group (7.83 ± 1.26%; *p* = 0.012). This finding reflects the association between metabolic disturbance severity and PCS development. In addition, the PCS group demonstrated slightly elevated levels of hs-CRP (*p* = 0.315) and IL-6 (*p* = 0.278), reflecting a link to inflammation and lower levels of vitamin D_3_ in individuals suffering from PCS (17.43 ± 8.26 vs. 21.89 ± 12.67 ng/mL; *p* = 0.091); however, these differences were not statistically significant due to high variability in PCS patients.

### COVID-19-related anamnesis of the study participants

More than 70% of patients without PCS reported mild COVID-19 disease severity. Patients with T2D and PCS were more frequently diagnosed with moderate and severe forms of COVID-19 infection. In general, the prevalence of moderate-to-severe forms of COVID-19 infection was 2-fold higher in the PCS group than in patients without PCS (56.8% vs. 27.2%, *p* = 0.161; Table 1).

Differences in the management of active COVID-19 infection were also observed. Patients with PCS reported higher rates of glucocorticoid prescriptions (36.4% vs. 13.6%; *p* = 0.055). In addition, patients with PCS more frequently received O_2_ therapy (27.3% vs. 9.1%; *p* = 0.089). The use of supplements such as zinc, vitamin D_3_, non-steroidal anti-inflammatory drugs (NSAIDs), and/or antibiotics was approximately on the same level and did not differ significantly between groups (Table 1).

### Relative telomere length analysis

**Figure 1:**
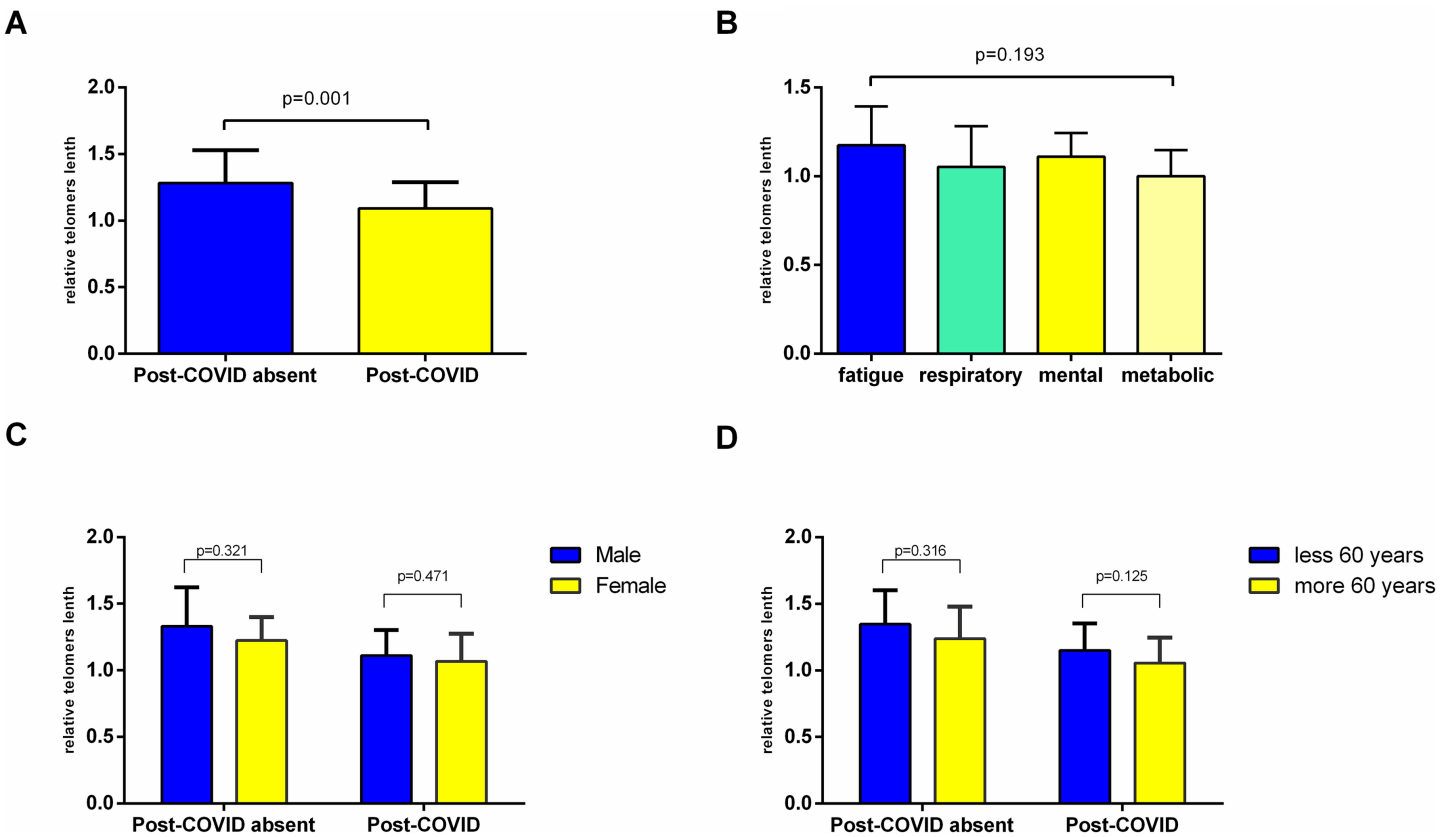
Comparison of the relative telomere length in study participants. Bar plots represent mean ± standard deviation. **(A)** Depending on PCS present; **(B)** difference between main clinical phenotypes of PCS; **(C)** gender-specific analysis; **(D)** age-dependent analysis. **(A,C,D)** Independent Student’s *t*-test; **(B)** one-way ANOVA was used for comparison. *p < 0.05.

In contrast to serum biomarkers, telomere length was closely associated with PCS. The results of our research showed significantly lower relative mean telomere length in patients with PCS as compared to those without it (1.09 ± 0.19 and 1.28 ± 0.24; *p* = 0.001; Figure 1A). This finding highlights the link between the development of PCS and the acceleration of biological aging mechanisms.

Patients of the main group were subdivided according to the following clinical phenotypes of PCS: fatigue and/or pain (*n* = 13), mental disorders (*n* = 10), cardiorespiratory symptoms (*n* = 12), and metabolic-associated symptoms (*n* = 9). The average length of telomeres was the shortest in patients who experienced worsening of metabolic-associated symptoms (1.00 ± 0.14). In patients with a predominance of fatigue, the mean relative telomere length in leukocytes was 1.17 ± 0.2; in mental and cardiorespiratory symptoms, it was 1.11 ± 0.13 and 1.05 ± 0.23. However, the relative telomere length did not differ significantly between clinical phenotype subgroups (*p* = 0.193; Figure 1B).

The gender-specific sub-analysis demonstrated shorter telomere length in women (Figure 1C). In women with a history of PCS, the relative telomere length was 1.06 ± 0.20, which was lower than the length in men, that is, 1.11 ± 0.19 (*p* = 0.471); however, these differences were not statistically significant. Among patients without a history of PCS, the relative length of telomeres in leukocytes was 1.22 ± 0.17 in women and 1.33 ± 0.29 in men (*p* = 0.321). In both groups, gender-specific differences were not significant (Figure 1C).

In another sub-analysis, we compared the relative telomere length in patients with T2D regarding age (Figure 1D). Naturally, the shortening of telomere length in patients over 60 years as compared to younger patients was demonstrated and was more pronounced in the PCS group (1.05 ± 0.19 vs. 1.15 ± 0.20; *p* = 0.125). Gender- and age-specific telomere shortening have important implications for understanding the biology of PCS and its potential underlying mechanisms.

Overall, these findings point to the importance of considering gender and age when investigating telomere dynamics and their implications for PCS pathophysiology, prognosis, and tailored therapeutic strategies.

### Association between telomere length and main parameters

Although there were no statistically significant differences in serum pro-inflammatory biomarkers between PCS patients and the comparison group, we found a significant association between telomere shortening and hs-CPR and IL-6 levels (Table 2). A univariate Pearson’s correlation analysis revealed an inverse correlation between relative telomere length and pro-inflammatory markers—IL-6 (*r* = −0.289; *p* = 0.049) and hs-CRP (*r* = −0.305; *p* = 0.044), especially in the PCS group. Additionally, in all T2D patients, telomere length correlated with age (*r* = −0.256; *p* = 0.038) and HbA1c (*r* = −0.265; *p* = 0.032).

**Table 2:** Correlation analysis between relative telomere length and other parameters.

Parameter	All patients (*n* = 66)	PCS absent (*n* = 22)	PCS present (*n* = 44)
Age, years	−0.256 (0.038)*	−0.376 (0.093)	−0.258 (0.090)
T2D duration, years	0.054 (0.669)	−0.052 (0.822)	0.090 (0.538)
HbA1c, %	−0.265 (0.032)*	−0.149 (0.520)	−0.161 (0.296)
Weight, kg	−0.077 (0.537)	−0.026 (0.911)	−0.137 (0.374)
BMI, kg/m^2^	−0.168 (0.178)	−0.195 (0.398)	−0.162 (0.293)
Waist circumference, cm	−0.059 (0.641)	−0.131 (0.570)	−0.092 (0.553)
Total cholesterol, mmol/L	0.148 (0.235)	0.158 (0.494)	0.194 (0.208)
Apolipoprotein B, g/L	0.211 (0.089)	0.160 (0.487)	0.271 (0.075)
Apolipoprotein A1, g/L	0.070 (0.578)	−0.067 (0.773)	0.033 (0.832)
Vitamin D_3_, ng/mL	0.002 (0.987)	−0.322 (0.155)	0.146 (0.344)
hs-CRP, mg/L	−0.196 (0.049)*	0.005 (0.984)	−0.305 (0.044)*
IL-6, pg/mL	−0.182 (0.143)	0.128 (0.581)	−0.289 (0.049)*

The data are presented as r (p); BMI, body mass index; SUs, sulfonylureas; ADD, anti-diabetic drugs; PCS, post-COVID-19 syndrome. Bold and * indicates significant changes.

In the univariate logistic regression model, telomere length was significantly associated with the presence of PCS (OR 0.016; 95%CI 0.001–0.262; *p* = 0.004).

### Assessment of telomere length as a potential marker for post-COVID-19 syndrome

Among the evaluated models, GBM emerged as the most effective; the best model reached an AUROC of 0.753. The least-performing model within the top 10 was DRF, with an AUROC of 0.672. Notably, the top 10 list comprised 7 GBM models, 1 DRF, 1 XRT, and 1 XGBoost, showcasing a gradual decline in AUROC values from the top-ranking model to the 10th-ranked one.

**Figure 2:**
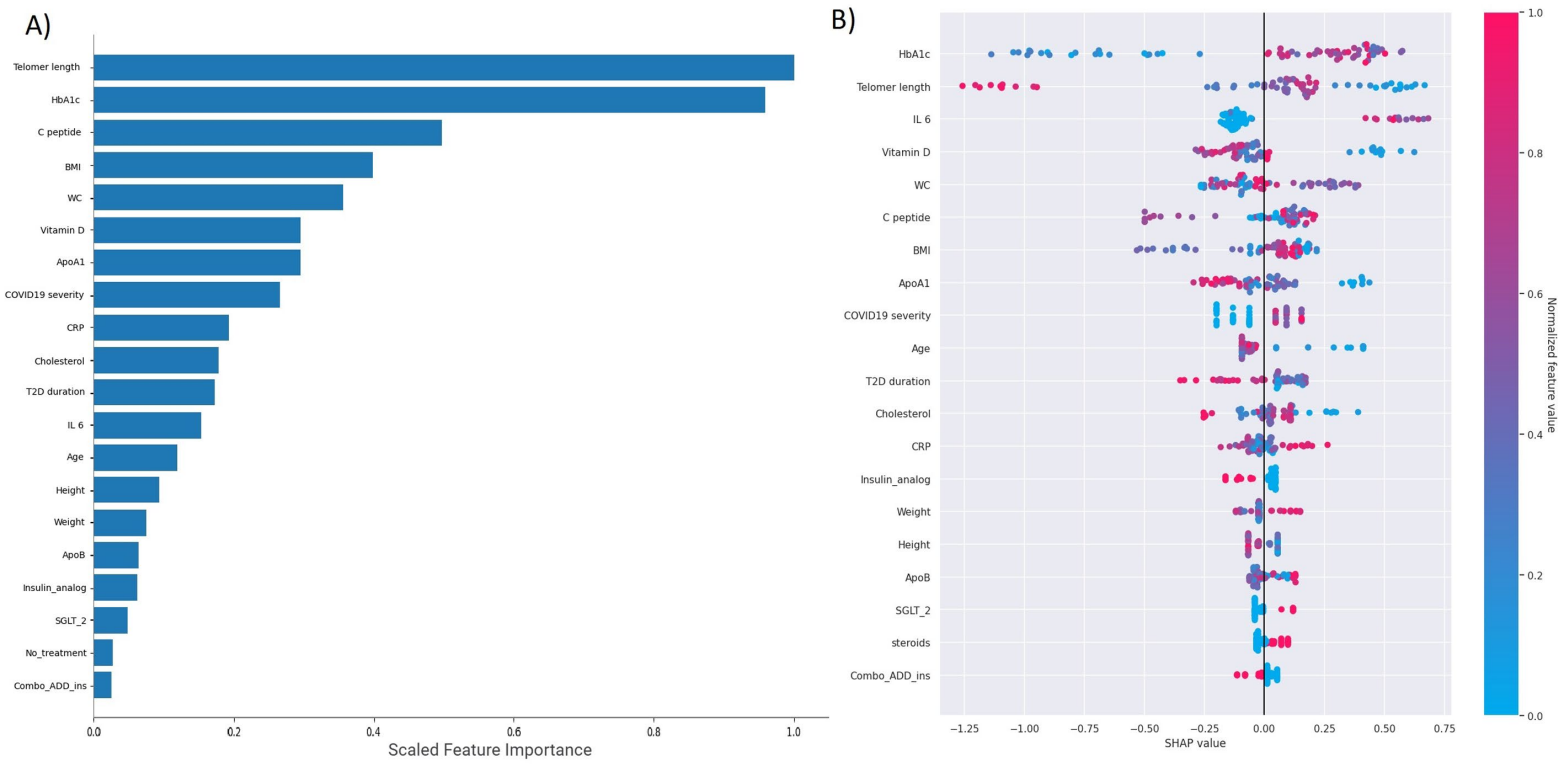
The top 20 features’ importance derived from permutation analysis **(A)** and SHAP **(B)** analysis within the most effective classification model. A total of 59 features were utilized in constructing a model aimed at identifying post-COVID-19-positive patients. The analysis encompasses the overall influence of each feature, focusing primarily on the top 20. It not only delineates the impact of these features but also elucidates how this impact varies. Each row within the figure corresponds to a specific feature, while the x-axis represents the SHAP value. Each data point signifies a sample, with colors closer to red indicating higher values and those closer to blue indicating lower values. For instance, telomere length emerges as a critical feature exhibiting a negative correlation with post-COVID-19 status. Thus, higher values of telomere length correspond to a lower likelihood of post-COVID-19 determination. **(A)** Displays the ranking of permutation feature importance for predicting PCS by the top model. Telomere length and HbA1c stand out as the most influential factors in PCS prediction. This significance is further emphasized when examining the feature importance of the top 10 models, as shown in Figure 3. In this analysis, the prominence of HbA1c and telomere length becomes even more pronounced. Conversely, vitamin D, waist circumference, and ApoA1 exhibited significantly weaker importance, while the remaining variables had even lower levels of significance. **(B)** Illustrates variables arranged in descending order of importance, with each patient represented by a dot on each variable line. The horizontal position of each dot indicates whether the effect of a variable is associated with a higher or lower likelihood of PCS. Variable-specific SHAP values greater than 0 suggest an increased risk of PCS.

**Figure 3:**
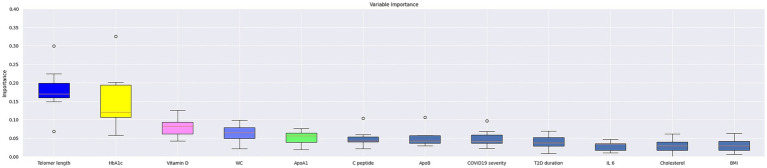
Permutation importance of the top features across the top 10 models for PCS identification. The permutation importance of each feature for every model was calculated and is shown in box plots.

To explore the relative importance of different variables associated with the presence of PCS, we used permutation feature importance and SHAP values. The best machine learning classification model identified HbA1c and telomere length as the features with the highest importance, followed by IL-6, vitamin D_3_, and waist circumference. The SHAP analysis indicated that lower HbA1c values were associated with a significantly lower likelihood of having PCS. Similarly, individuals with longer telomeres had a lower likelihood of being in the PCS group, while those with very short telomeres were more likely to have the syndrome (Figures 2, 3).

**Figure 4:**
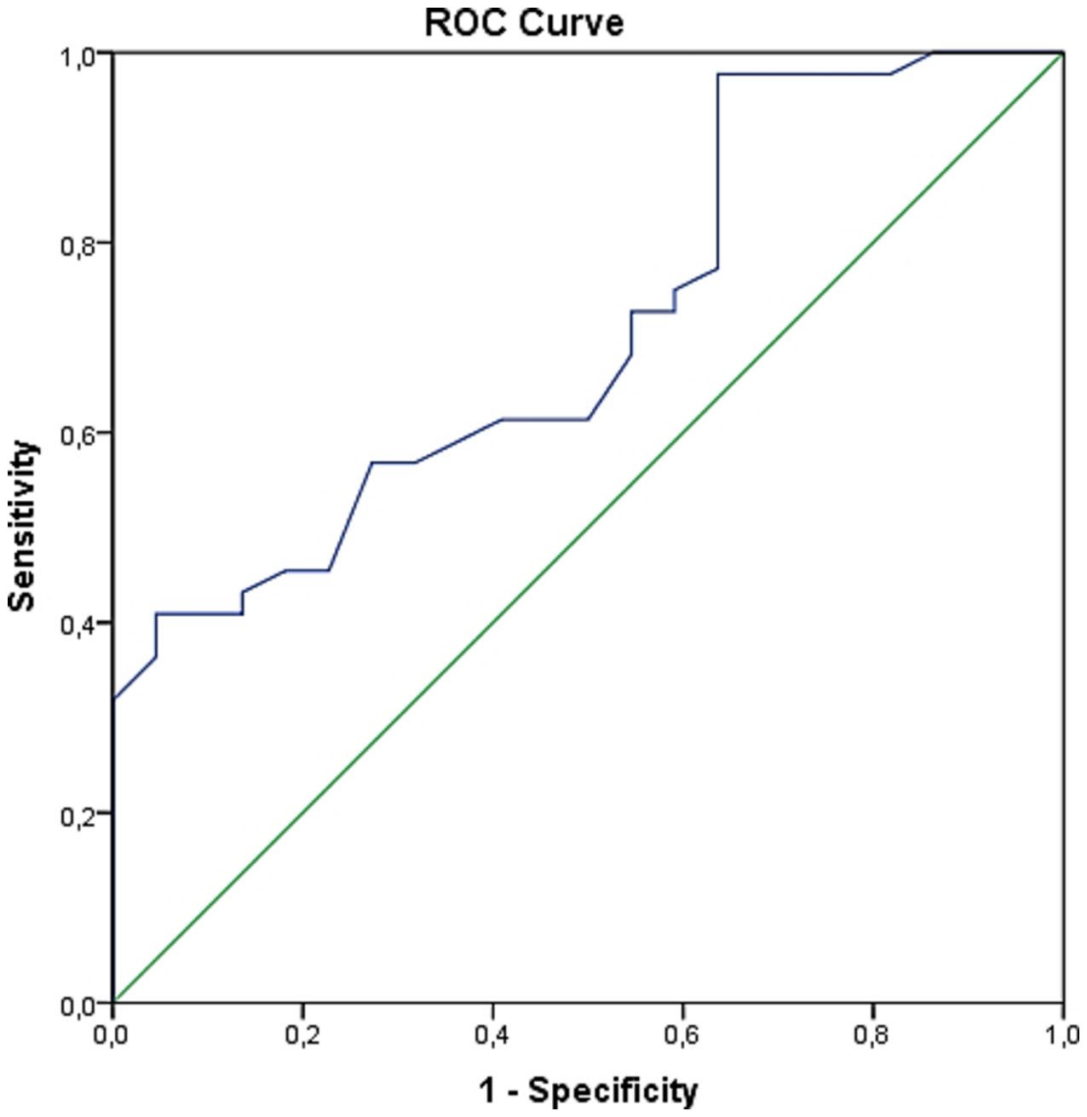
ROC curves used as a predictor of relative telomere length for PCS development in T2D patients.

To further assess the specific ability of telomere length to differentiate between patients with and without PCS, we performed an ROC analysis. This analysis yielded a statistically significant AUROC of 0.708 (95% CI 0.580–0.836; *p* = 0.006; Figure 4). At an exploratory cutoff value of ≤1.03 for telomere length, the specificity for identifying patients with PCS was 90.9%, and the sensitivity was 40.9%.

## Discussion

The results of the study demonstrated the close association between telomere length and PCS development in T2D patients. Our results showed a significant shortening of telomere length in patients with PCS, which allows us to support the fact of accelerated biological aging in the PCS population. Similarly, significant shortening of telomere length was observed in PCS as compared to COVID-19-free subjects (*p* < 0.0001) (37). In contrast, other authors found that shorter telomere length is associated with COVID-19 hospitalization but not with persistent PCS manifestations (29).

While previous investigations have established the predictive value of HbA1c for assessing COVID-19 severity, our study is among the first to underscore the significance of pre-COVID-19 HbA1c levels as a factor associated with the later presence of PCS in patients with T2D (38). Additionally, HbA1c together with telomere length has been identified as one of the strongest markers of biological age, further supporting the notion that accelerated biological aging could be a significant factor in PCS development (39).

Growing evidence has postulated that PCS appears to be more common among women, older adult individuals, and those with existing comorbidities and higher BMI (40). We observed shorter telomere length in women and patients older than 60 years in both groups. Previous reports also have shown accelerated biological aging and telomere attrition following SARS-CoV-2 infection (41, 42). Furthermore, relative telomere length was significantly lower in the women of reproductive age with COVID-19 than in the control group (*p* < 0.05) (43) and associated with a higher risk of mortality due to COVID-19 (44). The observed trend of shorter telomeres in women with PCS compared to men, although not statistically significant, is surprising, as women have longer telomere lengths (45). On the other hand, women may experience greater vulnerability to stress-induced telomere attrition (46). This may be linked to sex-based differences in hormonal regulation, immune response, and oxidative stress, which are known to influence telomere maintenance (47). Despite the lack of significant differences, these findings may highlight potential gender-related disparities in the biological impact of PCS, suggesting the need for further research to explore the role of sex hormones and other gender-specific factors in telomere dynamics.

Age-specific telomere shortening, particularly in patients over 60 years old, underscores the natural process of telomere attrition with aging and its potential exacerbation in the context of PCS. The near-significant difference in telomere length between older and younger PCS patients suggests that PCS may accelerate age-related telomere shortening. This finding could reflect a greater biological burden of PCS in older individuals, potentially due to the cumulative effects of inflammation, oxidative stress, or immune dysregulation, which are common features of both aging and PCS (48). Understanding these age-related changes could help identify high-risk groups and guide interventions targeting telomere preservation in older PCS patients.

Our data align with the previous study results. Several investigations conducted during the COVID-19 pandemic have also demonstrated a link between shorter telomeres and higher mortality (49). A strong inverse correlation between relative telomere length and COVID-19 severity has been described, with telomere shortening accelerating biological aging and telomere attrition after SARS-CoV-2 infection (41, 42, 50). In an ordinal regression model, T2D showed the strongest association with telomere length among various diseases. In the same study, telomere length shortening was called a biomarker of COVID-19 infection severity (51). According to Mendelian randomization (MR) analysis, shorter telomere length was associated with a higher risk of adverse COVID-19 outcomes, independently of other risk factors, including age (52). However, other analyses have not shown such a causal relationship (53, 54). Patients with shorter telomeres are known to have lower survival after COVID-19 (55). Persistent radiographic lung abnormalities have been observed in patients with shorter baseline telomere length (29). Accelerated aging may lead to a higher susceptibility to SARS-CoV-2 infection and severe coronavirus disease (56). Overall, recent data suggest that telomere length may serve as a biomarker associated with COVID-19 severity, adverse outcomes (such as hospital admission, need for critical care, and respiratory support), and mortality. However, there are limited data regarding the contribution of telomere length to PCS development. In this study, we evaluated the utility of telomere length as a variable associated with PCS and assessed its relationship with the clinical phenotype of PCS, gender, and biological age.

PCS presents one or more symptoms, including fatigue, dyspnea, chest and joint pain, and cognitive dysfunction, and others. Recent data on telomere length in patients with PCS are limited to several reports that demonstrate its shortening, mainly in pulmonary sequelae. Telomere shortening in patients with PCS was associated with a higher risk of developing and progressing pulmonary fibrosis, chronic obstructive pulmonary disease, and lung cancer (57). The results of the COVID-FIBROTIC cohort study showed the effects of SARS-CoV-2 on peripheral blood leukocyte telomere abrasion in patients after 12 months of infection and past bilateral pneumonia, as well as the presence of fibrotic changes in lung tissue. Specifically, 94.74% of patients were found to have shorter telomere length compared to age- and sex-matched controls 1 year after admission (58). Telomere length in alveolar type II (ATII) cells was studied in more detail, illuminating that their shortening is associated with long-term pulmonary fibrosis in PCS patients (30). The *RTEL1* gene has been identified, which encodes a helicase that regulates telomere length increase. The ultra-rare variant of this gene is considered a marker of evolutionary fibrotic changes in the lungs in the PCS phase (59). In our study, we were the first to analyze telomere length in terms of different PCS presentations. The shortest telomere length was observed in patients with cardiorespiratory symptoms, compared to those with fatigue/pain, mental disorders, and metabolic disorders.

Translating our data to clinical settings may require particular management of people with diabetes who suffer from COVID-19 or other respiratory infections at different stages of the infectious disease. Shortening of the telomere length in patients who recovered from COVID-19 may indicate that SARS-CoV-2 infection can cause telomere erosion in blood cells, especially in leukocytes (43). From a clinical point of view, hs-CRP and inflammatory cytokines, such as IL-6, may be important markers of COVID-19 severity and the development of PCS syndrome. We observed a mild negative correlation between relative telomere length and both pro-inflammatory markers. Previously, a negative correlation between C-reactive protein, neutrophil-to-lymphocyte ratio, and telomere length in patients with COVID-19 was also described (50).

On the other hand, inhibition of telomere shortening may be considered a potential target for the treatment of COVID-19 and the consequences of PCS. For this purpose, different strategies should be considered to effectively deal with this disease in the long term. In this regard, the role of several enzymes involved in telomere length regulation has recently attracted much attention (60, 61). Probably, a healthy lifestyle, regular physical activity, and a healthy diet will contribute to telomere elongation, better redox state, lower levels of inflammation, and the ability to have strong resistance to COVID-19 itself and long-term sequelae such as PCS (62).

Given the modest size of our dataset, constructing a generalized model for PCS prediction was not feasible. However, our analysis serves as a valuable resource for identifying key variables that warrant further investigation in future, larger studies. To this end, our application of permutation feature importance revealed a clear hierarchy of factors associated with the presence of PCS (Figure 3). Notably, telomere length and HbA1c demonstrated the strongest association with the syndrome. A secondary group of variables with a moderate association included vitamin D_3_, waist circumference, and ApoA1. A weaker, yet still observable, association was found for other factors such as C-peptide, ApoB, COVID-19 severity, T2D duration, IL-6, cholesterol, and BMI. These findings help prioritize targets for future longitudinal and interventional research.

### Limitations of the study

The findings of this study should be interpreted in the context of several key limitations. The primary limitation is the cross-sectional pilot design, which can establish association but does not allow for the inference of causality or prognostic relationships. Therefore, while our data suggest a link between telomere length, HbA1c, and PCS, we cannot conclude that these factors are predictive of the syndrome’s course.

Another significant limitation is the modest sample size. While our analysis was sufficient to suggest a preliminary association between telomere length and the presence of PCS, these findings require validation in larger, adequately powered cohorts to be confirmed.

These limitations underscore the need for further research. Future prospective cohort studies are needed, incorporating several time points for assessing telomere length with respect to patient outcomes, diabetes, and the course of PCS. Such studies should also include more patients and analyze telomere maintenance pathways, which may influence telomere length, alongside HbA1c levels. Finally, the exploration of relations between different metabolic circuits and signaling pathways in T2D combined with viral infection could shed light on telomere maintenance mechanisms, paving the way for future interventional studies and the development of tailored treatments.

## Conclusion

In this pilot study, we found that patients with T2D and PCS had significantly shorter telomere lengths and higher HbA1c levels than patients without PCS. These findings suggest that markers of accelerated cellular aging and altered glycemic control are associated with the presence of PCS in this population. Therefore, telomere length and HbA1c warrant further investigation in larger, longitudinal studies to determine their potential as biomarkers for risk stratification.

## Data Availability

The raw data supporting the conclusions of this article will be made available by the authors, without undue reservation.
